# Comparison of the Efficacy Between Regional Nerve Block and Ring Block as Local Anesthetic Techniques for Platelet-Rich Plasma Treatment

**DOI:** 10.7759/cureus.53901

**Published:** 2024-02-09

**Authors:** Sharanika A Nagaja, Rubin S John, Santhosh P Kumar, Murugesan Krishnan

**Affiliations:** 1 Oral and Maxillofacial Surgery, Saveetha Dental College and Hospitals, Saveetha Institute of Medical and Technical Sciences, Saveetha University, Chennai, IND

**Keywords:** local dental anesthesia, platelet-rich plasma, ring block, regional nerve blocks, postoperative pain, local analgesia

## Abstract

Introduction

Platelet-rich plasma (PRP), a solution of concentrated platelets, has been widely used to promote wound repair and tissue regeneration. In the treatment of pattern hair loss, platelets in PRP secrete an abundance of growth factors, including platelet-derived growth factor (PDGF), fibroblast growth factor(FGF), and many more, which stimulate and increase signaling molecules and accelerate cell proliferation. In the PRP treatment for hair regrowth, the supratrochlear nerve (STN) block and supraorbital nerve (SON) block are given to anesthetize the scalp up to the vertex except for the temporal region. The ring block is the common local anesthetic technique used by infiltrating local anesthetic agents around the target area. The primary objectives were to compare the pain and anesthetic success rates produced by regional nerve blocks and ring blocks.

Materials and methods

A sample size of 100 patients undergoing PRP treatment for hair regrowth were taken as the subjects for the study. Patients were allotted into two groups by randomization. Group 1 was given regional nerve blocks as the anesthetic technique used for local anesthesia, and group 2 was given ring blocks. In the study group, STN and SON blocks as the regional nerve blocks were given 2% lignocaine with 1:80000 adrenaline to anesthetize the area, and the PRP was injected from the anterior hairline up to the vertex of the scalp, not involving the occipital and temporal regions. In the control group, a ring block was given for the same procedure. Participants from both groups were assessed for the pain and analgesia caused by ring block and regional nerve blocks using the visual analog scale (VAS).

Results

A mean rank of 30.28 was observed for the regional nerve block technique, and a mean rank of 70.72 was observed for the ring block technique. A p-value of 0.00 that is <0.05 was observed, which shows there is a significant difference in the pain and the analgesia experienced by the subjects between the two groups, during and three hours after the procedure.

Conclusion

PRP is one of the most commonly used treatments for hair regrowth. The ring block is the common local anesthetic technique used for producing anesthesia, while regional nerve blocks are more effective in producing local anesthesia. This study proves that STN and SON blocks are better anesthetic techniques than the ring block technique for PRP treatment in hair growth.

## Introduction

In the field of hematology, platelet-rich growth factors often known as platelet concentrate, or platelet-rich plasma (PRP), were first reported in the 1970s. PRP is a word used by hematopathologists to refer to a high-platelet product used in thrombocytopenia treatment [1]. Proteins and growth factor concentrate made from centrifuged whole blood that has been stripped of its red blood cells constitute PRP. It is an autologous blood product that has been enhanced with platelets. These platelets contain bioactive proteins and growth factors that support angiogenesis and tissue regeneration [2]. Because PRP contains a high concentration of growth factors, it has been demonstrated in the domains of sports medicine and orthopedics to promote soft tissue and joint recovery [3]. PRP has been utilized to accelerate wound healing in a variety of medical specialties since the 1990s, including urology, gynecology, and oral and maxillofacial surgery. Moreover, it is utilized in reconstructive and face plastic surgery for soft tissue augmentation, wound healing, skin renewal, and hair development [4].

The process of PRP starts with a straightforward blood sample from the patient, and then the platelets are carefully separated and concentrated using centrifugation. The resulting PRP is then precisely injected into the desired area, be it joints, muscles, or skin, to start a series of reparative reactions. This powerful elixir is bursting with regeneration components [5]. Researchers published one of the first papers on PRP for androgenetic alopecia (AGA) in 2006, and they found that PRP-treated areas had a 15% higher follicular unit density hair yield than control areas [6]. This sparked curiosity, advanced the development of PRP technology for hair restoration, and accelerated its use in clinical settings. Chen et al. looked specifically at PRP for hair restoration in patients with AGA in a more recent systematic review study. Promising findings were obtained from the analysis of patient demographics, treatment frequency, hair count, and hair density after PRP therapy [7].

With minimal side effects, a thorough analysis of the current literature reveals a growing body of evidence supporting its safety profile. However, injections and local anesthetics are used in this PRP treatment. The two most used blocks for hair PRP are regional nerve and ring blocks [8,9]. For regional nerve blocks, PRP therapy frequently makes use of the supraorbital nerve (SON), supratrochlear nerve (STN), and auriculotemporal nerve blocks. The anterior, medial, and lateral scalp regions are innervated by the nerves that these blocks target, in that order. The ophthalmic division of the trigeminal nerve gives rise to the SON and the STN [10]. Both of them are readily accessible to neural blockage and go through the orbit above the orbital ridge [11]. By placing the needle slightly above the eyebrow across its medial border and injecting 1-2 ml of lidocaine, bupivacaine, or a combination of the two, the STN is blocked. The injection can be made here to anesthetize the SON, which is located about 2 cm lateral to the STN, or it can be made laterally using the same puncture used for the STN, using 1-2 cc of anesthetic [12]. Ring block anesthesia is the second technique used in this study. Using a ring block, a local anesthetic is injected around the scalp in a circular motion to provide regional anesthesia in 20-25 injections [13].

When receiving PRP therapy, it's critical to comprehend how to evaluate pain and how well these local anesthetic injections work. The aim of this study was to evaluate the efficacy of the ring block technique versus the regional nerve block technique as local anesthetic techniques for PRP treatment on the scalp for hair regrowth treatment. The primary objectives were to compare the pain and anesthetic success rates produced by regional nerve blocks and the ring block techniques.

## Materials and methods

Study design and setting

This prospective comparative study was done in the Departments of Oral and Maxillofacial Surgery of Saveetha Dental College and Hospitals, Saveetha Institute of Medical and Technical Sciences, Saveetha University, in Chennai, India, from June 2022 to May 2023. This study was approved by the Institutional Human Ethical Committee of Saveetha Dental College and Hospitals (approval number: IHEC/SDC/OMFS-2203/23/289).

Sample size

Considering α=5% and a power of 95, the sample size of 100 patients was calculated using G*Power with the help of a study done by Yang et al. in 2019 [14]. Given that this is a parallel-arm trial with an allocation ratio of 1:1, the patients were divided into two groups. 

Inclusion and exclusion criteria

All patients with a history of hair fall with diffuse thinning of hair and categorized under Norwood Grades 2 and 3, patients who needed PRP treatment for hair growth, and patients who were over 18 years old were included in the study. Patients with systemic conditions, patients who were not willing to participate in the study, and patients with complete hair loss were excluded from the study. After the patients were assessed for eligibility, they were sampled by the block randomization method and allocated into two groups. Group 1 was given a regional nerve block, and group 2 was given a ring block. 

Procedure

**Figure 1:**
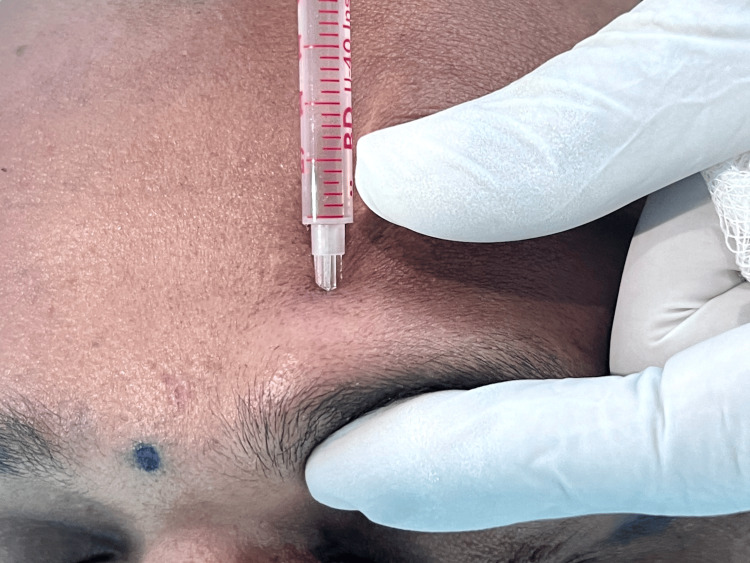
Landmark for supratrochlear nerve block

The patients were given an appointment and were explained about the procedure. For both groups, 10 mL of blood was drawn from the radial vein. Blood was centrifuged at 2400 rpm for 10 minutes and at 3600 rpm for 15 minutes. For group 1, the STN block was given slightly above the eyebrow across its medial border, injecting 2 cc of 2% lignocaine with 1:80000 adrenaline to anesthetize the area (Figure 1). SON block was given 2 cm lateral from the point of injection for the STN block using the same amount of local anesthesia, and the PRP was injected using insulin syringes from the anterior hairline up to the vertex of the scalp, not involving the occipital and temporal regions. Regional anesthesia requires two injections on each side and then takes five to seven minutes for onset of anesthesia. For group 2, the patients were given a ring block to anesthetize the same region using 6 cc of 2% lignocaine with 1:80000 adrenaline. This required 20-25 injections around the scalp and was very similar to infiltration. The treatment done was the same for both appointments, other than the type of block given for local anesthesia. Participants were assessed for pain perception and analgesia caused by the nerve blocks using the visual analog scale (VAS) [15] during the procedure and three hours after the treatment.

Statistical analysis

Statistical analysis was done using IBM SPSS Statistics for Windows, V. 23.0 (IBM Corp., Armonk, NY). Descriptive statistics were done to assess the median and percentage of the VAS scores. The normality test was assessed by the Shapiro-Wilk test. The Mann-Whitney U test was done to assess the differences in the pain score between the injection techniques with a statistical significance less than 0.05. 

## Results

**Figure 2:**
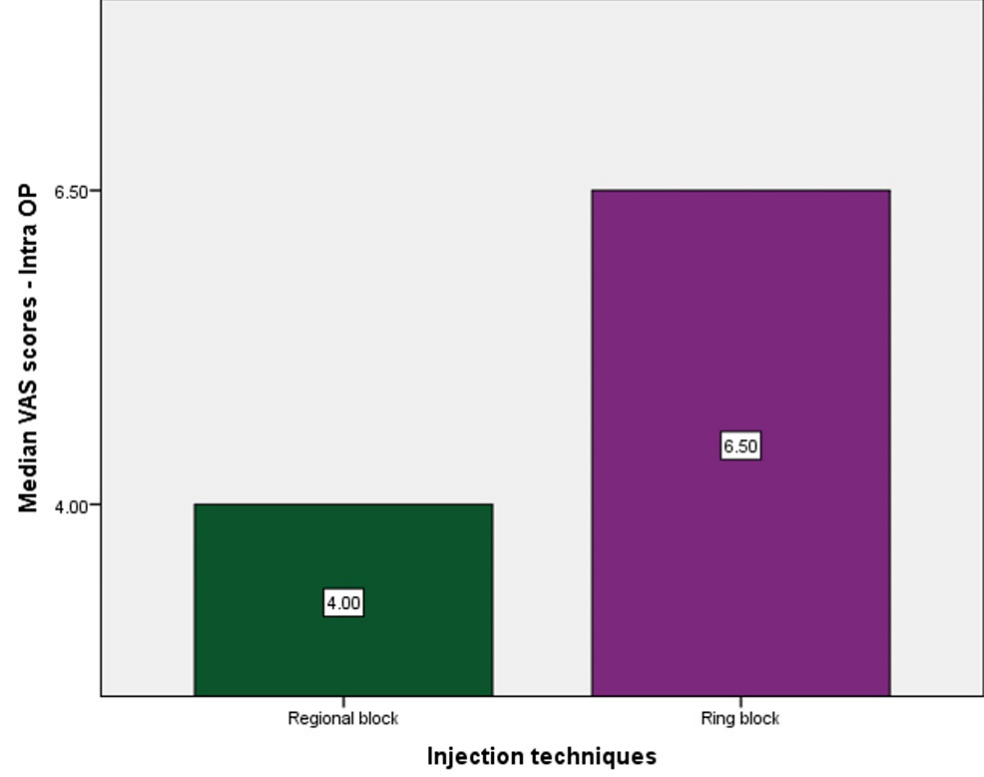
Median pain score perceived by the patients between both injection techniques during the procedure

**Figure 3:**
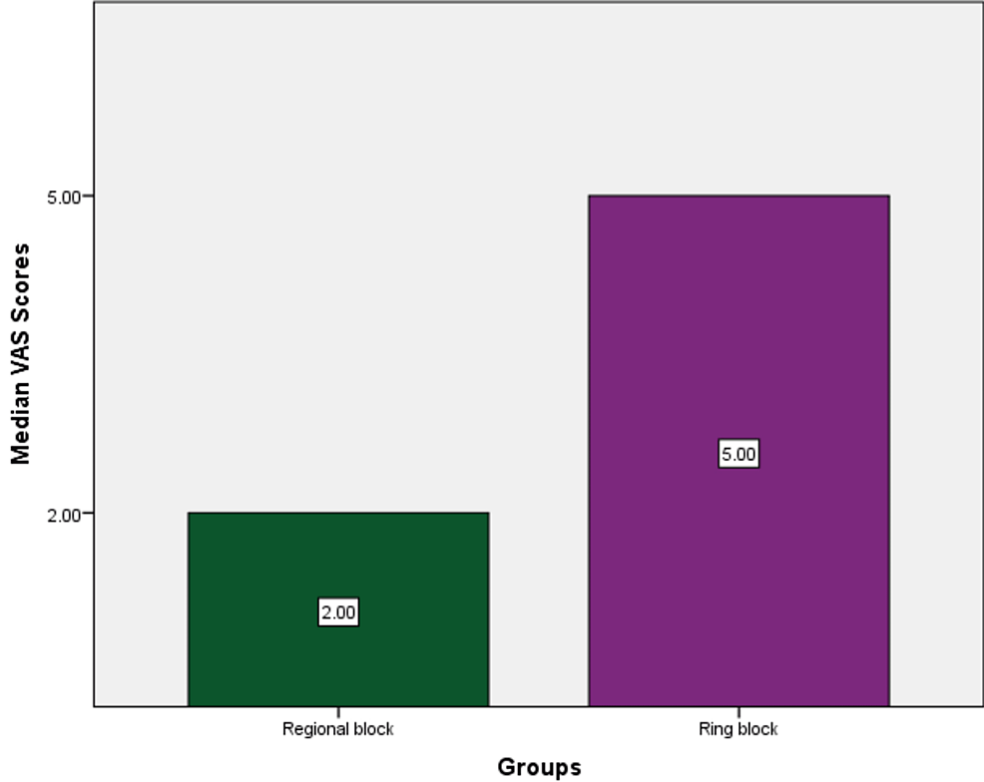
Median pain score perceived by the patients between both injection techniques three hours after the procedure

The study assessed the pain perceived by patients with two injection techniques used in PRP treatment for hair growth. This trial had 100 patients who participated in the study, and the pain was scored using the VAS, which was expressed as ordinal data. The median pain scores perceived by the patients for the regional nerve block were 4 and 2, and for the ring block, the median pain scores perceived by the patients were 6.5 and 5 during the procedure (Figure 2) and three hours after the procedure (Figure 3), respectively.

Results showed that the lowest individual score perceived by patients during the regional nerve block technique was 0 (6%) and the highest was 4 (4%). For the ring block technique, the minimum score was 1 (6%) and the maximum score was 8 (2%) (Table 1).

**Table 1:** Percentage of individual pain score perceived by the patients between both injection techniques VAS: visual analog scale; N: number; %: percentage

VAS scores	Regional nerve block N (%)	Ring block N (%)
0	3 (6)	0
1	14 (28)	3 (6)
2	18 (36)	3 (6)
3	13 (26)	4 (8)
4	2 (4)	13 (26)
5	0	15 (30)
6	0	10 (20)
7	0	1 (2)
8	0	1 (2)
Total	50	50

The Mann-Whitney U test compared the pain perceived with both injection techniques. It revealed that the pain perceived by patients during the regional nerve block was significantly less than that of the ring block and the difference in scores between groups was statistically significant (Table 2).

**Table 2:** Mann-Whitney U test showing the significant differences between the pain scores among the two techniques *: statistically significant

Groups	Mean rank	Sum of ranks	Mann-Whitney U value	P-value
Regional nerve block	30.28	1514	239	0.001*
Ring block	70.72	3536		

## Discussion

AGA is a chronic hair loss disorder that affects 40% of women and 80% of white men under the age of 70 years who have female pattern hair loss (FPHL) and male pattern hair loss (MPHL). Although hair loss can manifest in both genders as early as 18 years of age, the course of this condition varies significantly between them [16]. Although FPHL is characterized by diffuse thinning rather than complete baldness, hair loss in men typically follows well-defined patterns, as best illustrated by the Norwood and Hamilton scales. Finasteride and minoxidil are two of the current drugs that the United States Food and Drug Administration (FDA) has confirmed and approved for AGA [17,18]. A systematic review by Adil and Godwin revealed that minoxidil, finasteride, and low-level laser light therapy are useful in encouraging hair growth in men with AGA and that minoxidil was useful in promoting hair growth in women with AGA [19]. PRP has longer-lasting advantages when these effects are reversible. PRP's effects were evaluated for a year in a 2020 study by Gentile and Garcovich, and the results showed an increase in hair density and count [20]. In order to stimulate hair follicles and encourage hair growth, PRP therapy involves injecting PRP into the scalp. This can reverse AGA and FPHL, but several injections are needed. Therefore, in order to reduce patient discomfort and guarantee a smoother treatment experience, local anesthesia is crucial. PRP therapy frequently uses two types of local anesthesia: ring block and regional nerve block [21].

A regional nerve block is a procedure used to block pain perception in a particular part of the body by injecting local anesthetics into targeted nerves. Before undergoing PRP injections, the region is effectively made numb by regional nerve blocks, which target the nerves supplying sensation to the scalp [22]. However, this regional anesthesia doesn't cover the temporal and occipital regions. It is a sensitive anesthetic technique, but it covers a large area and is more focused and exact. A minimal amount of local anesthetic is needed when using a regional nerve block, and there is less chance of systemic side effects [23]. It requires a lesser number of pricks and lasts longer. However, the main disadvantage of this block is it is more painful to administer even with a smaller number of injections [24]. But in spite of all the disadvantages, the regional nerve block may also offer analgesia following surgery. According to multiple studies, in the 24-48 hours after craniotomies, the prevalence of moderate to severe postoperative pain increased from 60% to 80% [25,26].

On the other hand, ring block, by obstructing every sensory nerve that supplies the scalp, attempts to anesthetize the whole region. But as it is very similar to infiltration, it is not very painful during administration, and the onset is relatively quicker which is five to seven minutes. But it covers less area, and the anesthesia effect does not last long [27]. While studies have demonstrated that interfollicular injections can be used in hair PRP treatments without requiring a nerve block, the patient will still experience some discomfort during the procedure [28,29]. The present study proved that performing PRP therapy under local anesthesia provides a much better experience for the patient.

Limitations of the study

Firstly, there are only 100 patients involved in the study. Also, only two injection techniques were compared. Future studies should be conducted with a larger sample size and should be comparing various injection techniques. The choice between regional nerve block and ring block for PRP therapy depends on various factors, including patient preference, physician expertise, and the desired level of anesthesia.

## Conclusions

This study's results illuminate a crucial aspect of pain management in PRP treatment for hair growth, specifically comparing two injection techniques: regional nerve block and ring block. The findings unequivocally reveal a significant difference in pain scores between the two injection techniques, with the regional nerve block causing notably less discomfort than the ring block. The reduced pain associated with regional nerve blocks aligns with the broader objective of maximizing patient comfort during medical interventions. As PRP treatment gains popularity in aesthetic and regenerative medicine, addressing pain concerns becomes crucial to ensure positive patient outcomes and overall satisfaction. The study acts as a foundation for further investigations and advocates for a nuanced consideration of patient comfort in the planning and execution of PRP procedures.
